# A Bite So Bad: Septic Shock Due to Capnocytophaga Canimorsus Following a Dog Bite

**DOI:** 10.7759/cureus.14668

**Published:** 2021-04-24

**Authors:** Michelle-Ashley Rizk, Nicholas Abourizk, Kinjal P Gadhiya, Panupong Hansrivijit, John D Goldman

**Affiliations:** 1 Department of Internal Medicine, University of Pittsburgh Medical Center Pinnacle, Harrisburg, USA; 2 Division of Infectious Diseases, University of Pittsburgh Medical Center Pinnacle, Harrisburg, USA

**Keywords:** severe sepsis, septic shock, capnocytophaga, capnocytophaga canimorsus, dog bites, animal bite, critically ill patients

## Abstract

Infection by *Capnocytophaga canimorsus (C. canimorsus)*, a Gram-negative rod found in the normal oral flora of canine and feline species and transmitted through bites or scratches, is a rare phenomenon. Infections most commonly occur in alcoholics and immunocompromised patients. In this report, we describe the case of a middle-aged male with a history of alcohol abuse who presented with septic shock and multisystem organ failure following a suspected dog bite. The patient was a 59-year-old Caucasian male with alcohol abuse disorder who initially presented with encephalopathy and lethargy. The patient had scratches and multiple healing wounds, with a mottled appearance on his extremities. According to his wife, the patient had been playing aggressively with his dog at home. On admission, he was febrile, tachycardic, and saturating in the 80s on ambient air. His extremities rapidly developed diffuse purpura and dry gangrene of all digits along with the tip of his nose and genitals. The patient developed septic shock and multisystem organ failure. Blood cultures initially grew Gram-positive cocci and Gram-negative rods for which broad-spectrum antibiotics were initiated. Follow-up blood cultures were positive for *C. canimorsus* and the antibiotic regimen was adjusted accordingly. His condition continued to deteriorate. His family opted for comfort measures only and he died soon after. Common sequelae of *C. canimorsus* infection include septic shock with multisystem organ failure, disseminated purpuric lesions, hypotension, encephalopathy, and acute renal failure. As seen in our patient, *C. canimorsus* infection should be considered in such patients, particularly if there is a recent history of an animal bite. Prompt initiation of appropriate treatment is essential to improve patient prognosis.

## Introduction

*Capnocytophaga canimorsus (C. canimorsus)* is found in the normal oral flora of most domestic canines and felines [1] and is transmitted to humans by bites, licks, and scratches [1]. Infections are most commonly seen in men between the ages of 50-70 years, especially those who are immunocompromised, asplenic, or alcoholic [1]. It is considered to be of low virulence in healthy people; however, in certain cases, the infection can lead to severe complications and even death [1]. Common sequelae of *C. canimorsus* infection include septic shock with multisystem organ failure, disseminated purpura, hypotension, encephalopathy, and acute renal failure [1]. Even with the administration of adequate antimicrobial therapy, *C. canimorsus*-induced septicemia can progress to debilitating disease or septic shock with high mortality rates [2]. In this report, we present a case of a 59-year-old male with concomitant history of alcohol abuse who presented with septic shock and multiorgan failure secondary to *C. canimorsus* following several incidences of dog bites and scratches on his upper and lower extremities after play-fighting with his own canine.

## Case presentation

A 59-year-old Caucasian male with a medical history of alcohol abuse disorder (consumption of an average of 30 beers daily) presented with progressively worsening encephalopathy and malaise of one week's duration. His wife reported that he had experienced subjective fevers for two days prior to the presentation. The patient had scratches and multiple healing wounds on his upper and lower extremities along with a mottled appearance that was cool to the touch. According to his wife, the patient had developed these wounds from playing aggressively with his dog on daily basis over the past few weeks. His dog would often scratch, bite, and lick him.

On admission, the patient was encephalopathic. In the emergency department, he was febrile with a temperature of 39.3 °C, tachycardic with a heart rate of 147 beats per minute, normotensive with blood pressure 133/94 mmHg, and was saturating in the 80s on room air. He was intubated for airway protection, started on broad-spectrum antibiotics with vancomycin, cefepime, and metronidazole, and was admitted to the intensive care unit.

The patient's laboratory test results were as follows: white blood cell count of 2,000/L, hemoglobin of 14.5 g/dL, platelet count of 20,000/uL, prothrombin time of 19.2 seconds, an international normalized ratio (INR) of 1.7, albumin of 2.3 g/dL, lactic acid of >12 mmol/L, procalcitonin of 304 UG/L; his C-reactive protein was 21.8 mg/dL, HIV antibody was non-reactive, viral hepatitis panel was found to be negative, serum ethanol level was <10 mg/dL, aspartate transaminase was 1,393 U/L, alanine transaminase was 289 U/L, ammonia was 65 mol/L, lipase was within normal limits, acetaminophen and salicylate levels were negative, and troponin was 0.18 ng/mL (peaked at 1.36 ng/mL). D-dimer was >7,000 ng FEU/mL, and fibrinogen was 165 mg/dL; the peripheral smear was negative for schistocytes but showed numerous bacteria within the white blood cell cytoplasm. His creatinine level and creatine kinase activity were elevated. He required continuous renal replacement therapy for acute renal failure. An echocardiogram showed a newly reduced left ventricular ejection fraction of 10-15%.

**Figure 1:**
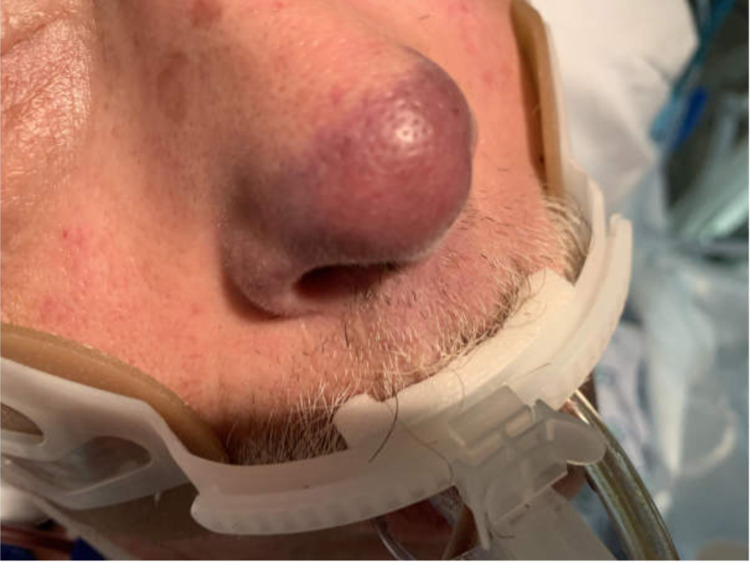
Necrosis of the tip of the nose caused by Capnocytophaga canimorsus infection

**Figure 2:**
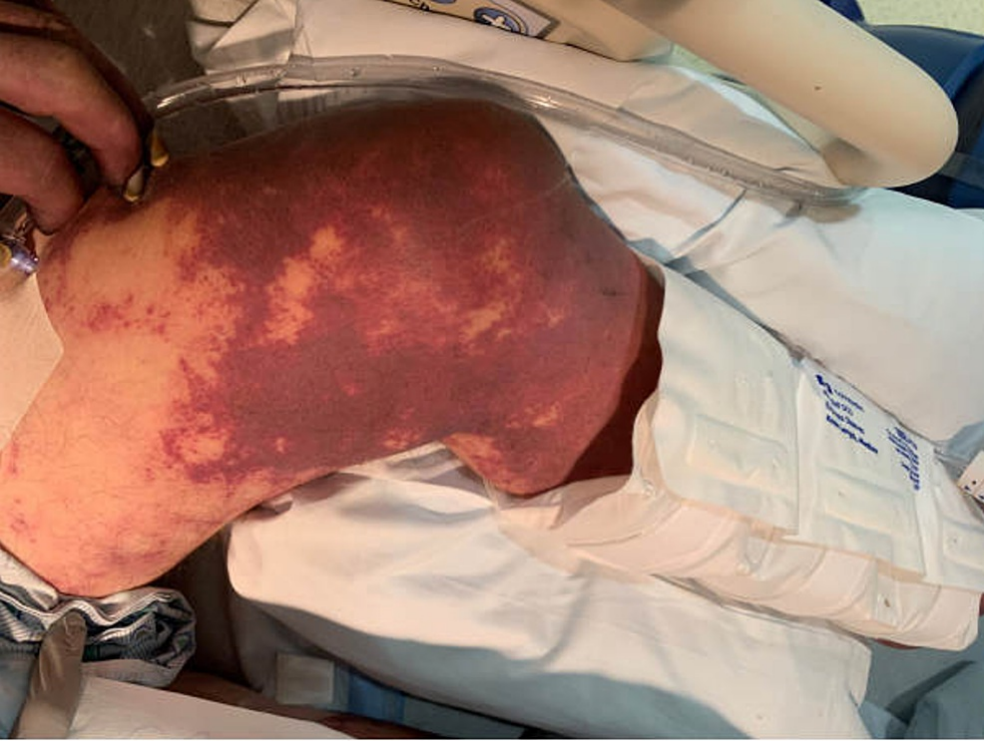
Purpura fulminans present on the left lower extremity in the setting of Capnocytophaga canimorsus infection

**Figure 3:**
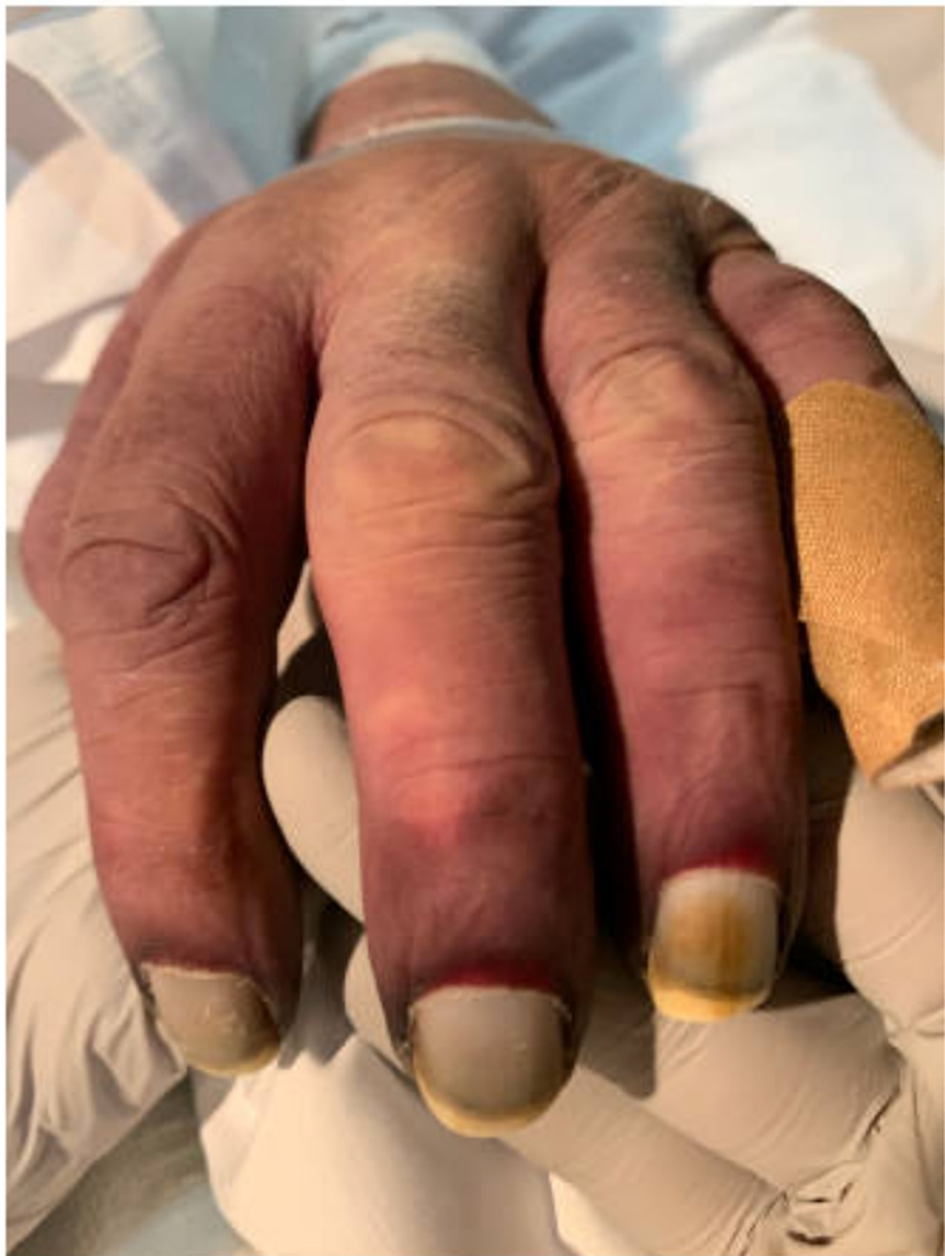
Dry gangrene of the fingertips of the left hand in the setting of Capnocytophaga canimorsus-induced septic shock

During hospitalization, his upper and lower extremities were found to have numerous scratches and small crusted healing wounds with a mottled appearance. He quickly developed diffuse purpura and dry gangrene of all digits, along with the tip of his nose and genitals over a period of four days (Figures 1, 2, 3). The patient developed septic shock with multisystem organ failure eventually.

Blood cultures initially grew Gram-positive cocci and Gram-negative rods in one out of four bottles. He was initially treated with vancomycin, cefepime, and metronidazole. Infectious Diseases was consulted. Given the patient's history of alcohol abuse and dog bites, *C. canimorsus* was high on the differential. The antibiotic regimen was changed to intravenous vancomycin, clindamycin, doxycycline, and piperacillin/tazobactam. The initial blood cultures eventually grew *C. canimorsus*, approximately four days after specimen collection, which was confirmed by DNA sequence analysis. The antibiotic cocktail was then narrowed to ampicillin/sulbactam and clindamycin. Despite all these measures and extensive intensive care, the patient’s condition continued to deteriorate and progressed to multiorgan failure. Eventually, all care was withdrawn as per the patient's family's wishes, and the patient passed away.

## Discussion

Etiology, epidemiology, and risk factors

Eighty-one of the 484 documented cases of this type of infection reported in the literature between 1961 and 2014 turned out to be fatal. Its incidence is on the higher side in the United States, The Netherlands, Denmark, and France [3]. The risk factors include splenectomy, alcohol use, liver cirrhosis, smoking, and malignancy. Patients with functional asplenia or those with a previous history of splenectomy are at risk of overwhelming infections, especially with encapsulated organisms such as *C. canimorsus*, which can progress rapidly and can be fatal. It is important to note that infections are more common in immunocompromised patients but can also occur in immunocompetent patients [3-6].

Clinical features and complications

Human infection occurs by dog bite or, rarely, cat bite or by the licking of wounds by these animals. The incubation period ranges from one to eight days [1,7]. The initial presentation generally includes fever, fatigue, gastrointestinal symptoms, shortness of breath, followed by purpura [1,5]. Subsequently, it leads to sepsis, disseminated intravascular coagulation, thrombotic thrombocytopenic purpura, purpura fulminans, cellulitis, symmetrical gangrenes, meningitis, endocarditis, and multiorgan failure [3-6]. However, there have been cases where fulminant central nervous system manifestations were observed in the setting of immunocompetent states [1,8]. Rare presentations include cholecystitis, paravertebral abscess, Sweet’s syndrome (acute neutrophilic dermatosis with fever), and Waterhouse-Friderichsen syndrome [1]. As observed in this patient, significant elevation of hepatic enzymes, prothrombin time, and ammonia along with the reduction of serum albumin level could suggest concomitant acute liver failure, which carries a remarkably high mortality rate. However, the patient's mental status or asterixis, a hallmark of acute liver failure, could not be evaluated as he was intubated and sedated.

Microbiology

*C. canimorsus, C. cynodeginia,* and *C. canis* are considered to be part of dog and cat mouth flora. *C. canimorsus* and C. *cynodeginia* are found to be zoonotic pathogens due to catalase and oxidase activities, and *C. canis* is not as virulent in humans due to the lack of oxidase activity [9]. *C. canimorsus* is a Gram-negative, capnophilic, and facultatively anaerobic organism that is part of normal flora in dogs and cats [3]. It is slow-growing and flourishes with carbon dioxide enrichment [1]. Certain identifiable characteristics include positive test results for oxidase, catalase, arginine dihydrolase, and o-nitrophenyl-ß-d-galactopyranoside, as well as negative reactions for urease, nitrates, and indole [1,3].

Pathogenesis 

*C. canimorsus* is protected by a lipooligosaccharide and capsule. This causes human serum to be ineffective in protecting against this bacterium and offers relative resistance to phagocytosis by macrophages [1,3]. The factors associated with its virulence include resistance to complements, production of catalase that allows bacteria to survive inside the phagocytes, lack of flagella that facilitates travel through the tissue to reach the bloodstream, and possessing of sialidase that helps to get amino sugar nutrients from the host cells [1,3]. Given splenic macrophages in the red pulp and liver sinusoid-containing Kupffer cells have innate immunity of early phagocytosis against *C. canimorsus,* even though antibody production usually takes some days to fight against the bacteria, splenectomized patients lack early phagocytosis mechanisms and therefore tend to develop early septic shock and have shown a high case fatality rate [3].

Diagnostic aspects

Being a slow-growing bacterium, it can take up to 14 days for *C. canimorsus* to be detectable on cultures, and hence the clinical history and physical signs are significant for early management [7]. Blood, body fluids (such as the cerebrospinal fluid), and, less often, tissue from infected sites of the bite can be collected and examined in Gram stain and culture [1]. The gold standard for diagnosing *C. canimorsus* infection in humans has been molecular tests based on polymerase chain reaction (PCR) and sequencing, particularly detecting 16S ribosomal RNA gene sequencing [1,10,11].

Treatment

Despite having such a resilient defense mechanism, *C. canimorsus* is relatively susceptible to ß-lactam antibiotics, third-generation cephalosporins, doxycycline, clindamycin, and chloramphenicol or carbapenems. The addition of a ß-lactamase inhibitor is preferred to ß-lactam antibiotics alone due to the increasing incidence of ß-lactamase-producing bacteria, especially when choosing oral antibiotic therapy [12]. The preferred intravenous agents include clindamycin, doxycycline, cefuroxime, and meropenem [13]. There is typically low sensitivity to aminoglycosides, trimethoprim, and aztreonam [7].

Swift recognition of *C. canimorsus* infection and prompt initiation of antibiotics is paramount. Treatment duration can vary depending on the complications that are encountered. Infective endocarditis would warrant four to six weeks of antibiotic therapy. Infection with bone or joint involvement would require a longer course of approximately six to eight weeks. The treatment of *C. canimorsus* with meningitis could take 14-21 days or longer, depending on the clinical scenario [1]. One case report has documented the use of plasmapheresis and leukapheresis, which led to patient recovery [6].

Preventative measures

Amoxicillin/clavulanic acid is the first-line antibiotic prophylaxis after dog or cat bite, and doxycycline or clindamycin can be considered in the setting of penicillin allergy [1,7,13].

## Conclusions

Unfortunately, surveillance for *Capnocytophaga* spp. is not generally performed, likely due to its rarity. Early recognition of the cause of the infection is important to prevent the deterioration of the patient's condition due to associated high mortality. Empirical antibiotic therapy with penicillin in combination with a ß-lactamase inhibitor should be initiated early on, especially in patients with liver disease and a history of alcohol abuse presenting with dog or cat bites. For patients with purpura fulminans, digital dry gangrene, immune thrombocytopenic purpura, or disseminated intravascular coagulopathy, a high index of suspicion should be maintained for *Capnocytophaga* spp. infection and they should be promptly tested. Prompt initiation of appropriate treatment is essential for favorable patient outcomes.
